# Human papillomavirus infection and immunohistochemical expression of cell cycle proteins pRb, p53, and p16^INK4a^ in sinonasal diseases

**DOI:** 10.1186/s13027-015-0019-8

**Published:** 2015-08-04

**Authors:** Yukashi Yamashita, Masahiro Hasegawa, Zeyi Deng, Hiroyuki Maeda, Shunsuke Kondo, Asanori Kyuna, Sen Matayoshi, Shinya Agena, Takayuki Uehara, Hideaki Kouzaki, Takeshi Shimizu, Taro Ikegami, Akira Ganaha, Mikio Suzuki

**Affiliations:** Department of Otorhinolaryngology, Head and Neck Surgery, Graduate School of Medicine, University of the Ryukyus, Okinawa, 903-0215 Japan; Department of Otorhinolaryngology, Head and Neck Surgery, Zhujiang Hospital, Southern Medical University, Guangzhou, China; Department of Otolaryngology, Head and Neck Surgery, Shiga University of Medical Science, Otsu, 520-2192 Japan

**Keywords:** Human papillomavirus, Cell cycle protein, Inverted papilloma, Malignant transformation, Integration, Viral load, Sinonasal disease

## Abstract

**Background:**

We aimed to clarify the possible role of human papillomavirus (HPV) infection in the malignant transformation of sinonasal inverted papilloma (IP).

**Methods:**

Subjects comprised 32 patients with chronic rhinosinusitis (CRS), 17 with IP, 5 with IP and squamous cell carcinoma (IP + SCC), and 16 with primary sinonasal SCC. HPV presence, viral loads, and physical status were investigated using polymerase chain reaction. Retinoblastoma (pRb), p53, and p16^INK4a^ gene products were investigated by immunohistochemistry.

**Results:**

HPV DNA was detected in 6.3 % of cases with CRS, 29.4 % with IP, 40 % with IP + SCC, and 25 % with SCC. IP cases had significantly higher HPV presence than CRS cases (*p = 0.04*). High-risk HPV-16 was the most frequently encountered subtype (10/13, 76.9 %). HPV-16 viral loads varied from 2.5 to 7953 *E6* copies/50 ng genomic DNA. Patients in the SCC and IP + SCC groups had significantly higher viral loads than those in the IP and CRS groups (*p < 0.01*). All SCC and IP + SCC patients with HPV-16 demonstrated mixed-type integration, whereas 4 of 5 HPV-16 patients in the IP and CRS groups showed episomal type infection (*p = 0.04*). Positivity to pRb was found in 78.1 % of CRS, 35.3 % of IP, and 68.8 % of SCC cases. The presence of HPV DNA negatively correlated with pRb expression in SCC (*p* = 0.029) and IP (*P* = 0.049) groups. Although 62.5 % of SCC cases exhibited p53 positivity, only 5.9 % of IP, and no CRS cases were positive. Regardless of HPV status, p16^INK4a^ positivity was frequently detected in IP cases (82.4 %), less in SCC (12.5 %) cases, and was not detected in the CRS group. Neither the IP nor SCC cohorts showed any correlation between HPV presence and the expression of either p53 or p16^INK4a^.

**Conclusions:**

HPV infection was more frequent in the IP, IP + SCC, and SCC groups than the CRS group. Higher viral loads and integration observed in the IP + SCC and SCC groups, and an inverse correlation between HPV presence and positive pRb indicated that persistent infection and integration play a part in tumorigenesis and malignant transformation in certain IP cases. However, p16^INK4a^ is not a reliable surrogate marker for HPV infection in IP.

## Background

A variety of benign and malignant tumors may arise in the sinonasal cavity. Of these, inverted papilloma (IP), a benign neoplasm, has unique clinical characteristics; of note are its high rates of recurrence and malignant transformation. According to several systematic reviews and meta-analyses, the malignant transformation rate of IP is estimated to be approximately 10 % [3, 17, 20].

Human papillomavirus (HPV) infection in the sinonasal tract is estimated to be present in 37.8 % of IPs [32] and 27 % of squamous cell carcinomas (SCCs) [33]. Although a significant number of these tumors are infected with HPV, especially high-risk HPV types, whether HPV is involved in the malignant transformation of IP or in the pathogenesis of SCC has not been confirmed, as its mechanism and role in malignant transformation remain obscure. There are several of reports on the clinical relationship between IP and HPV infection. Two studies by Beck et al. found that 63 % of IP cases were positive for HPV DNA, and the presence of HPV sequences predicted the recurrence of inverted papilloma [4, 5]. Moreover, these studies found that patients with HPV types 16 or 18 have a higher rate of associated malignancy than patients with HPV 6 or 11 [5]. A systematic review subsequently confirmed that the presence of HPV was significantly associated with the likelihood of IP recurrence [18]. However, there continues to be discussion over whether there is a significant correlation between the development of malignant transformation in IP and HPV types [11, 16, 20].

In our previous study, viral loads and physical status of HPV were examined using fresh frozen samples of IP, SCC, and inflammatory mucosa from the sinonasal tract to clarify the clinical importance of HPV in IP. HPV genomes were detected in 46.1 % of IPs, 27.3 % of SCCs, and 7.6 % of the inflammatory group, respectively [10]. Because the IP group showed significantly higher HPV positivity rates than the inflammatory group, it was concluded that HPV infection is involved in the pathogenesis of IP, and a high viral load and integration of HPV may play important roles in malignant transformation.

The retinoblastoma (pRb), p16^INK4a^ and cyclin D1 genes are components of the pRb cell cycle control pathway. The active hypophosphorylated form of pRb binds and blocks the action of the transcription factor, inhibiting the transition from G1 to S phase. Cyclin D1 stimulates the phosphorylation of pRb by associating itself with cyclin-dependent kinases (CDKs). Binding of p16^INK4a^ to CDK 4 and 6 blocks their association with the D-type cyclins. Another well-known cellular tumor suppressor, p53, is involved in processes such as cell cycle progression, DNA repair, chromatin remodeling, differentiation, apoptosis and senescence. HPV-mediated tumorigenesis is mainly due to the activities of two viral oncoproteins, E6 and E7. HPV E6 can induce the degradation of p53 by direct binding to the ubiquitin ligase E6AP, inhibit p53-dependent signaling upon stress stimuli, and contribute to tumorigenesis [19, 27, 36]. The viral E7 protein binds to and inactivates pRb, activating E2F independent of cyclin dependent kinase. The functional inactivation of host pRb by HPV E7 protein results in overexpression of p16, making p16 a reasonable surrogate marker for the presence of high-risk human papillomavirus.

Several reports concerning cell cycle protein expression in IP, IP + SCC, and SCC have been published [1, 6, 14, 19, 21–23, 25, 27, 28, 36, 37]. However, the simultaneous evaluation of HPV infection and cell cycle protein expression using IP, IP + SCC, and SCC samples, using chronic rhinosinusitis (CRS) as a control, has not yet been reported. Moreover, it is very difficult to compare results because immunohistochemical methods of evaluation differ among studies. The aim of this study was to identify the HPV infection and immunophenotypic features in the malignant transformation of inverted papilloma. In the present study, additional cases of IP, IP + SCC, SCC, and CRS were recruited and HPV status (presence, viral load, and physical status) and cell cycle proteins related to HPV infection— retinoblastoma (pRb), p53, and p16^INK4a^ gene products—were investigated to reinforce our assumptions from the previous study.

### Ethics, consent and permissions

The study protocol was approved in advance by the Institutional Review Board of the University of the Ryukyus. This study was conducted to conform to the principles of the Declaration of Helsinki.

### Consent to publish

We have obtained consent to publish histologic pictures from the participants.

## Results

1. Patient characteristics (Table 1)

**Table 1:** Patient profiles

	IP	IP with SCC	SCC	CRS
Number of cases	17	5	16	32
Sex, n (%)				
Male	11	2	14	22
Female	6	3	2	10
Age (years)				
Mean	57	60	60	45
Range	44–76	55–72	40–82	17–81
≤50, n (%)	7	0	5	19
>50, n (%)	10	5	11	13
Krouse classification				
T1				
T2	3			
T3	13			
T4	1	5		
T classification				
T1				
T2			1	
T3			5	
T4			10	
N classification				
N0			10	
N1, N2, or N3			6

According to Krouse classification [17] of IP, T2 was observed in 3 patients, T3 in 13, and T4 in 1. The T4 case had a massive extension to the pterygoid fossa with no malignant lesions. The observation period after our surgeries for the IP group ranged from 18 to 98 months (median 43 months). At the first visit to our clinic, 7 patients had previous history of sinus surgery for IP and 2 cases (11.8 %) had recurrence after surgery.

Metachronous SCC occurred in 3 cases and synchronous SCC in 2 cases of the IP + SCC group. The periods between the initial treatments for IP to malignant transformation in the 3 metachronous SCC cases were 5 months, 6 years, and 9 years. Patients in the IP + SCC group received surgery and subsequent radiotherapy as adjuvant treatments for cancerous lesions. Although 1 case recurred after surgery combined with radiotherapy in the IP + SCC group, salvage endoscopic resection for the recurrent lesion was performed successfully and the lesion has not recurred for 9 years. The patient had HPV-16 infection with mixed integration.

2) HPV detection (Table 2)

**Table 2:** HPV presence, HPV type, viral load, and physical status of HPV-16

Groups	HPV presence	High-risk HPV (numbers)	HPV-16 viral load (E6 copies/50 ng genomic DNA)	E2/E6 of HPV-16	Integration type
SCC (n = 16)	25.0 %, 4 of 16	HPV-16, 3; HPV-18, 1	35	0.12	mixed
			79	0.1	mixed
			594	0.85	mixed
IP + SCC (n = 5)	40.0 %, 2 of 5	HPV16, 2	1524	0.65	mixed
			7953	0.67	mixed
IP (n = 17)	29.4 %, 5 of 17	HPV16, 3; HPV33, 2	2.5	1	episomal
			24.2	1	episomal
			74	1	episomal
CRS (n = 32)	6.3 %, 2 of 32	HPV16, 2	5.1	0.12	mixed
			6.8	1	episomal

HPV genomes were detected in 5 of the 17 patients in the IP group (29.4 %, Table 2), compared with 4 of the 16 patients (25.0 %) in the SCC group, 2 of the 5 patients (40 %) in the IP + SCC group, and 2 of the 32 patients (6.3 %) in the CRS group. The IP group showed a significantly higher HPV presence than the CRS group (*p = 0.04*, Fisher’s exact test).

The HPV types detected in the present study were high-risk types only (i.e. HPV-16, HPV-33, and HPV-18) and multiple HPV infections were not detected. Of the 13 HPV patients, HPV-16 was present in 10, therefore viral loads and physical status of HPV-16 were investigated in the positive samples. Viral loads varied from 2.5 to 7953 *E6* copies/50 ng genomic DNA. Patients in the SCC and IP + SCC groups had significantly higher viral loads than the IP and CRS groups (*p < 0.01*, Mann–Whitney U test). In addition, all patients with HPV-16 infection in the SCC and IP + SCC groups demonstrated mixed type integration, whereas 4 of the 5 patients with HPV-16 infection in the IP and CRS groups showed episomal type infection (*p = 0.01*, chi-square test).

3) Expression of pRb, p53, and p16^INK4a^ by immunohistochemistry (Table 3)

**Table 3:** Expression of cell cycle proteins

			p16^INK4a^		p53		pRB	
Groups	HPV presence		(+)	(−)	(+)	(−)	(+)	(−)
SCC	25.0 %	HPV (+), n = 4	1	3	2	2	1	3
(n = 16)		HPV (−), n = 12	1	11	8	4	10	2
IP + SCC	40.0 %	HPV(+), n = 2	2	0	1	1	0	2
(n = 5)		HPV(−), n = 3	0	3	0	3	0	3
IP	29.4 %	HPV (+), n = 5	4	1	0	5	0	5
(n = 17)		HPV (−), n = 12	10	2	1	11	6	6
CRS	6.3 %	HPV(+), n = 2	0	2	0	2	1	1
(n = 32)		HPV(−), n = 30	0	30	0	30	24	6

**Fig. 1:**
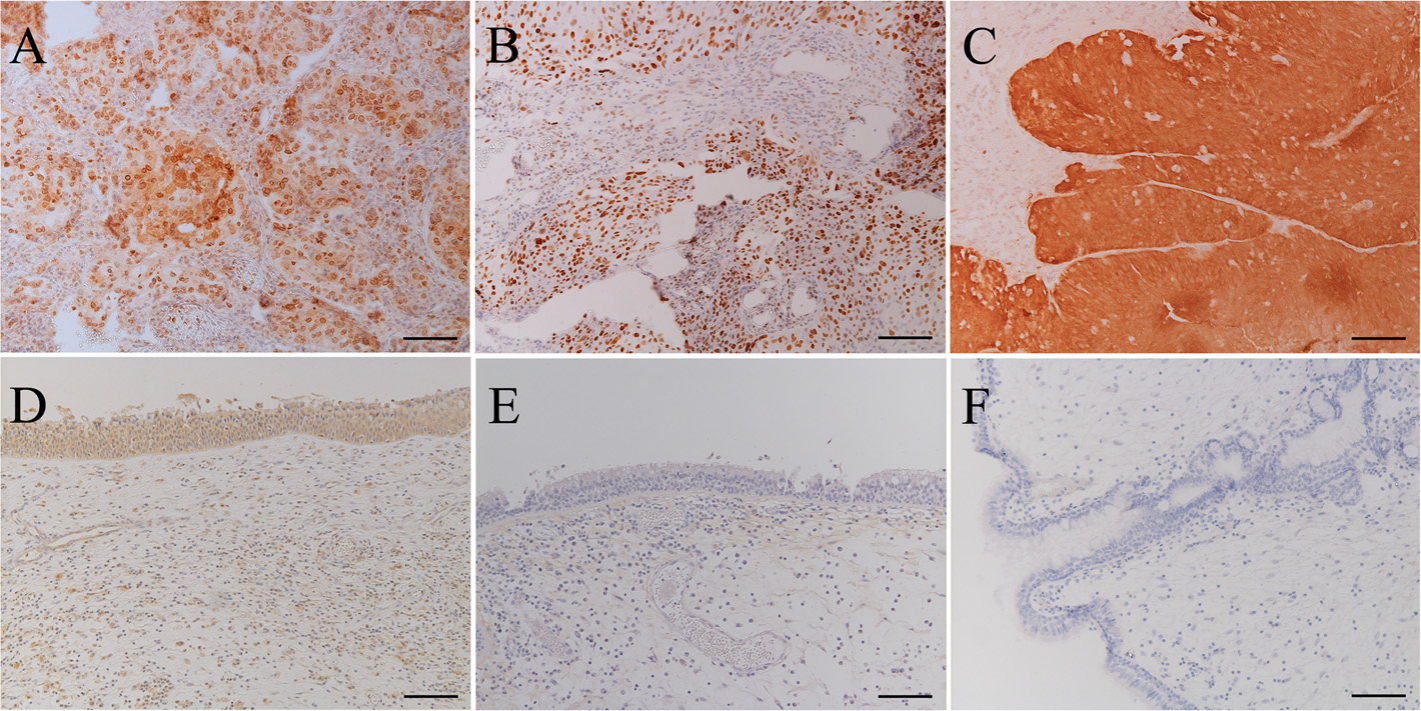
Representative immunohistochemistry results of pRb, p53, and p16^INK4a^. **a**: pRb immunohistochemistry of SCC without HPV infection. Bar = 100 μm. **b**: p53 immunohistochemistry of SCC part of inverted papilloma with SCC. The case showed HPV-16 positive. Bar = 100 μm. **c**: p16^INK4a^ expression in SCC with HPV-18 positivity. Tumor cells showed strong immunoreaction to p16^INK4a^ in this case. Bar = 100 μm. **d**: pRb expression in chronic rhinosinusitis. This case was HPV negative. Epithelial cells showed diffuse pRb immunoreaction. Bar = 100 μm. **e**: p53 expression in chronic rhinosinusitis. This case was also HPV negative. Epithelial cells showed no p53 immunoreaction. Bar = 100 μm. **f**: p16^INK4a^ expression in chronic rhinosinusitis. This case was HPV negative. The epithelial cells showed no p16^INK4a^ immunoreaction. Bar = 100 μm

Figure 1 shows the representative cases and expression of pRb, p53, and p16^INK4a^. The expression of pRb was observed in 78.1 % of the CRS group (Fig. 1d), 35.3 % of the IP group, none of the IP + SCC group, and 68.8 % of SCC group. The presence of HPV DNA showed negative correlation with pRb expression in the SCC group (*p* = 0.029, chi-square test). A similar relationship between the presence of HPV DNA and pRb expression was observed in the IP group (*p* = 0.049, chi-square test).

The expression of p53 was observed in 10 of the 16 patients (62.5 %) in the SCC group and 1 of the 5 patients (20 %) in the IP + SCC group, whereas it was expressed rarely in the IP (5.9 %) and CRS (0 %, Fig. 1e) groups (Table 3). The expression of p53 in the SCC and IP groups was not significantly related to HPV infection (Table 3).

**Fig. 2:**
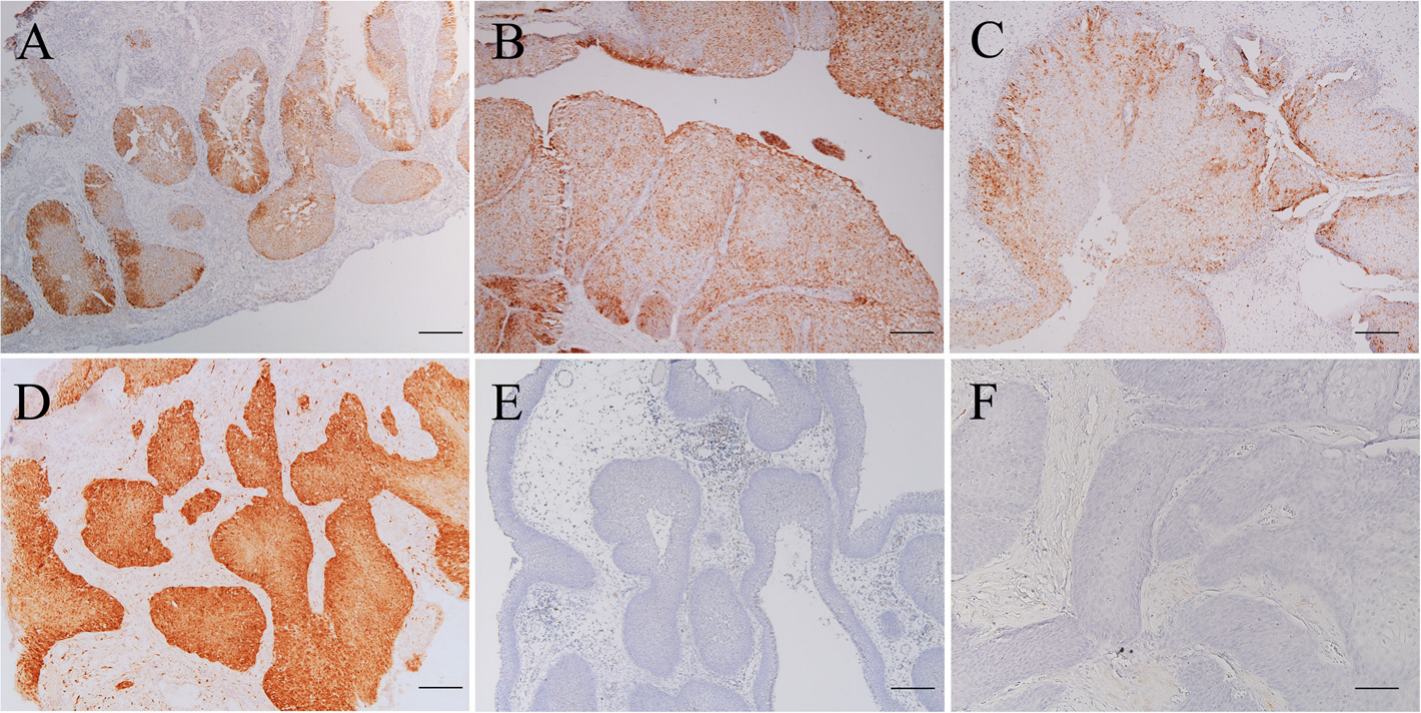
p16^INK4a^ immunohistochemistry in various cases. **a**: Inverted papilloma with positive p16^INK4a^ reaction. The case had HPV-16 infection with episomal form. Bar = 200 μm. **b**: Inverted papilloma with positive p16^INK4a^ expression. Human papillomavirus genome was not identified in this case. Bar = 200 μm. **c**: Inverted papilloma with positive p16^INK4a^ reaction. The case became squamous cell carcinoma later (Fig. 2d). The expression of p16^INK4a^ was more prominent in the basal layer than in the surface layer in inverted papillomas. HPV-16 with mixed integration was detected in this case. Bar = 200 μm. **d**: Metachronous squamous cell carcinoma that recurred after primary surgery for inverted papilloma (**c**). The invasive carcinoma cells displayed stronger p16^INK4^ expression compared with the inverted papilloma (**c**). HPV-16 was also detected with mixed integration. Bar = 200 μm. **e**: Inverted papilloma with negative p16^INK4a^ reaction. This case had HPV-33 infection. Bar = 200 μm. **f**: Inverted papilloma with negative p16^INK4a^ reaction. The human papillomavirus genome was not identified in this case. Bar = 200 μm

The expression of p16^INK4a^ was significant in the IP group at 82.4 % compared with 12.5 % in the SCC group and no expression (Fig. 1f) in the CRS group (both *p < 0.001*, chi-square test). HPV infection in the IP group was not related to p16^INK4a^ expression (Table 3). The case with HPV-33 infection in Fig. 2e showed no expression of p16^INK4a^, and expression was absent in 2 cases without HPV infection (Table 3, Fig. 2f).

In the IP + SCC group, 16^INK4a^ expression was observed in only patients with HPV infection. However, in the SCC group, 1 of the 4 patients with HPV infection showed p16^INK4a^ expression. Cases of p16^INK4a^ positivity in the SCC (Fig. 1c) and IP + SCC groups (Fig. 2d) showed strong p16^INK4a^ immunoreactivity compared with the IP group (Fig. 2a-c). Interestingly, a metachronous IP + SCC case with HPV infection showed relatively low expression of p16^INK4a^ when in IP only (Fig. 2c), whereas intense p16^INK4a^ expression was found in IP + SCC (Fig. 2d).

## Discussion

We previously reported that HPV infection is involved in the pathogenesis of IP and that high viral loads and integration of HPV may play important roles in malignant transformation [10]. In the present study, HPV infection was high in IP, IP + SCC, and SCC groups compared with the CRS group. According to a meta-analysis of HPV presence in sinonasal lesions, HPV was detected in 7.0 % of normal sinus mucosa, 4.1 % of nasal polyps, and 38.8 % of papillomas [32]. Furthermore, HPV was detected in 65.3 % of exophytic papillomas, 37.8 % of inverted papillomas, and 22.5 % of cylindrical cell papillomas [32]. According to a systematic review and meta-analysis of HPV prevalence in head and neck cancer, HPV was detected in 27.0–29.3 % of SCCs in sinonasal cancer [13, 33]. In the present study, the prevalence of HPV in CRS, IP, and SCC groups was consistent with previous reports [32, 33]. Although high-risk HPV types were detected in inflammatory diseases as well as in the IP and SCC groups, significant mixed type integration was observed in the SCC and IP + SCC groups. In addition, higher HPV-16 viral loads were detected in the SCC and IP + SCC groups, compared with the IP and CRS groups. These findings suggest that persistent infection and the integration of high-risk HPV types play important roles in the malignant transformation of IP.

Once HPV integration occurs, it leads to disruption of the HPV *E2* gene, which controls the expression of the oncogenes *E6* and *E7* by binding to and repressing their viral promoter, resulting in their abnormal expression [2]. E6 binds to the wild-type p53 via an adenosine triphosphate-dependent step, which leads to p53 degradation and inactivity. The E7 oncoprotein binds to and functionally inactivates pRB, which controls the crucial G1–S phase transition. Functional inactivation of pRb by E7 is known to induce up-regulation of p16^INK4a^ expression [15, 29].

In the present study, pRB expression was frequently observed in CRS, unlike p53, while expression was significantly less in the IP and SCC groups. In contrast, Altavilla et al. reported that all IP cases expressed both pRb and p16^INK4a^, regardless of HPV infection [1]. However, most of the cases in that study carried low-risk HPV types, whereas in the present study, all HPV cases carried high-risk types. The inverse correlation between HPV presence and pRb expression in the IP and SCC groups indicated that HPV infection in IP and SCC affects cell cycle protein expression, which suggests that HPV infection plays a role in tumorigenesis and malignant transformation.

Although the wild-type p53 is known to be involved in the negative regulation of cell growth, the mutant p53 promotes tumor formation through loss of growth suppression. Since the p53 antibody reacts to both wild- and mutant-type p53 proteins, it is impossible to distinguish between them using formalin-fixed, paraffin-embedded (FFPE) samples. However, the accumulation of p53 to levels detectable by immunohistochemistry is associated with p53 mutations [30]. In the present study, p53 expression was observed in only 1 patient in the IP and CRS groups, whereas the SCC group showed high p53 expression regardless of HPV presence. This finding suggests that the mutant p53 is rarely expressed in IP and CRS. Mirza et al. reported that the majority of HPV-positive cases did not express p53, possibly because of proteolytic degradation and elimination [20]. On the contrary, Schwerer et al. reported that the overexpression of p53 in inverted papilloma compared with normal nasal mucosa [28]. The range of p53 expression varies from zero to approximately one-third in IP [1, 19, 23, 37], whereas relatively high rates of expression are found in IP + SCC [19, 22, 37]. Lin et al. also reported that low expression of p16^INK4a^ and positive staining for p53 are important characteristics in IP + SCC compared with IP [19]. Although the reasons for these discrepancies among the p53 positivity rates between the studies are not clear, different antibodies and evaluation methods might have affected the results.

Expression of the tumor suppressor p16^INK4a^ has been proposed as a surrogate marker for HPV infection [7]. Overexpression of p16 is thought to reflect the presence of biologically active HPV infection. However, there are several contradictory reports on the value of p16^INK4a^ as a biomarker. Smith et al. found no concordance between p16^INK4a^ expression and HPV detection in 20 % of head and neck cancers [31], possibly due to transcriptionally inactive infection or an alternate pathway of p16^INK4a^ activation [34]. In our previous study, the diagnostic sensitivity of p16^INK4a^ overexpression was 53.2 % for the detection of HPV DNA in HNSCC, and was considerably better in oropharyngeal SCC at 80 % [8]. In the present study, p16 ^INK4a^ overexpression was identified in only 1 of the 4 SCC cases with HPV, but in more than 80 % of IP cases. These findings are consistent with previous reports [1, 19]. Although the mechanisms behind the high prevalence of p16^INK4a^ in IP are unknown, these results revealed that, in contrast to OPSCC, p16^INK4a^ immunoreactivity is not a surrogate marker for HPV infection in IP.

In summary, the abovementioned immunohistochemical characteristics in specimens from patients with HPV infection in the IP, IP with SCC, and SCC groups were similar to other HPV-related tumors. These immunohistochemical findings and current PCR-based results suggest that HPV infection is involved in inducing malignant transformation of IP through alteration of cell cycle protein expression.

## Conclusions

HPV infection was more frequently observed in IP, IP + SCC, and SCC groups than in the CRS group. The higher viral loads and integration observed in the IP + SCC and SCC groups and the inverse correlation between HPV presence and positive pRb in immunohistochemistry indicated that persistent HPV infection and integration are involved in tumorigenesis and malignant transformation in certain IP cases. However, p16^INK4a^ is not a reliable surrogate marker for HPV infection in IP.

### Subjects and methods

The subjects in the present study consisted of 17 patients with IP (IP group), 5 with IP associated with SCC (IP + SCC group), 16 with primary sinonasal SCC (SCC group), and 32 with chronic rhinosinusitis (CRS group). Surgical treatments were performed on all patients in the IP and IP + SCC groups. Specimens from 17 patients in the IP group, 1 in the IP + SCC group, 15 in the SCC group, and 32 in the CRS group were collected and stored in liquid nitrogen until analysis. FFPE samples were used for the analysis of only 4 of the 5 cases from the IP + SCC group, and fresh frozen samples were used for the analysis of all other cases in the CRS, IP, and SCC groups, and 1 case in the IP + SCC group.

1. Patient characteristics.
  The clinical features in each group, such as age, sex, and tumor stage, were reviewed from medical records.
2. Detection of HPV genome and identification of HPV types.

Fresh frozen samples were analyzed for HPV and HPV types as previously reported [9]. A Gentra Puregene tissue kit (Qiagen, Maryland) was used to isolate DNA from the specimens according to the manufacturer’s specifications. For the extraction of DNA from the IP-SCC and SCC FFPE samples, 3 10 μm-FFPE sections were placed in a microcentrifuge tube and mixed with 0.5 mL of DEXPAT kit (Takara, Otsu, Japan). After incubation at 100 °C for 10 min, the tube was immediately centrifuged at 12,000 rpm for 10 min at 4 °C. The supernatant was collected carefully, and 10 μL, including the extracted DNA, was used as a template for a 50 μL polymerase chain reaction (PCR) reaction according to the manufacturer’s protocol. The presence and integrity of the DNA was verified in all samples by PCR β-globin gene amplification using the primers PC04 and GH20 [26]. Negative controls with water and positive controls with the DNA of HPV-16-positive CaSki cell line were included in each amplification series.

The presence of HPV DNA was analyzed by PCR using the general consensus primer sets GP5+/GP6+ and MY09/11 [9]. DNA samples negative for GP5+/GP6+ or MY9/11 PCR were re-amplified in a nested PCR using the GP5+/GP6+ primer pair as previously described [9], which can increase the sensitivity of HPV detection. After purification of positive PCR products, sequence analysis was performed on an ABI PRISM 3130xl Genetic Analyzer (Applied Biosystems, CA). The sequences obtained were aligned, and compared with those of known HPV types available from the GenBank database using the BLAST program.

To investigate HPV-16 viral loads and physical status, quantitative real-time PCR using HPV-16 DNA-positive samples was performed to measure the quantities of the *E6* and *E2* genes of HPV-16 with the ABI Prism 7300 Sequence Detection System (Applied Biosystems) and TaqMan PCR Master Mix II (Roche Molecular Systems, Foster City, CA). The procedures, including experimental conditions, primers and TaqMan probes, were identical to a previous report [9]. Two standard curves for the *E6* and *E2* genes were generated by amplification of serial 10-fold dilutions (10^1^, 10^2^, 10^3^, 10^4^, 10^5^, and 10^6^ viral copies) of a plasmid pB-actin carrying the complete HPV-16 early region (Addgene, Inc., Cambridge, MA). For cellular DNA quantification, an external standard curve was generated using known serial dilutions (0.3, 3, 30, and 300 ng) of human genomic placental DNA (Sigma-Aldrich, St. Louis, MO), and *β-globin* was amplified as described by van Duin et al. [35]. The amount of DNA (in ng) was calculated by plotting the C_t_ (threshold cycle) values against the logarithm of the standard curve. The relative viral load was identified by calculating the copy numbers of specimen *E6* in 50 ng cellular DNA. The physical status of HPV-16 was determined according to a previously described method [24]. The amount of integrated *E6* was calculated by subtracting the copy numbers of *E2* (episomal) from the total copy numbers of *E6* (episomal and integrated). Ratios of *E2* copy number/total *E6* of <1 indicate the presence of both integrated and episomal forms (mixed-type integration). An *E2/E6* ratio nearly equal to 1 indicates predominance of the episomal form, whereas a ratio of 0 indicates the presence of only the integrated form.

3) Immunohistochemistry for pRb, p53, and p16^INK4a^.

Serial 4 μm-thick sections from FFPE samples were deparaffinized in xylene and hydrated in a graded series of alcohol. Epitope retrieval was performed by heating at 95–99 °C for 10 min in Tris/EDTA buffer (pH 9.0). Endogenous peroxidase activity was quenched by incubating the sections in 3 % hydrogen peroxide and 15 mM sodium azide for 5 min. The sections were subsequently incubated overnight at 4 °C with primary monoclonal mouse anti-pRb antibody (1:2000; LifeSpan BioSciences, Seattle WA) for pRb, primary monoclonal mouse anti-p53 antibody (1:500; Progen Biotech GmbH, Heidelberg, Germany) for p53 staining, and primary monoclonal mouse anti-p16^INK4a^ antibody (MTM Laboratories AG, Heidelberg, Germany). After extensive washing in phosphate-buffered saline, the slides were incubated for 30 min at room temperature with a horseradish peroxidase-conjugated goat anti-mouse secondary antibody (MTM Laboratories). Immunolabeling was visualized by incubation in 3-3′-diaminobenzidine and stained slides were counterstained with hematoxylin.

Cases were considered pRb-, p53-, or p16^INK4a^-positive when intense nuclear and/or cytoplasmic reactivity was present. Positive expression was defined as pRb and p53 staining in more than 25 % [12], or p16^INK4a^ staining in 40 %, of 2000 tumor cells [8].

## Abbreviations

CRS: Chronic rhinosinusitis
FFPE: Formalin-fixed, paraffin-embedded
HPV: Human papillomavirus
IP: Inverted papilloma
IP + SCC: Inverted papilloma with squamous cell carcinoma
PCR: Polymerase chain reaction
pRb: Retinoblastoma gene product
SCC: Squamous cell carcinoma
